# Med5(Nut1) and Med17(Srb4) Are Direct Targets of Mediator Histone H4 Tail Interactions

**DOI:** 10.1371/journal.pone.0038416

**Published:** 2012-06-05

**Authors:** Zhongle Liu, Lawrence C. Myers

**Affiliations:** Department of Biochemistry, Dartmouth Medical School, Hanover, New Hampshire, United States of America; Texas A&M University, United States of America

## Abstract

The Mediator complex transmits activation signals from DNA bound transcription factors to the core transcription machinery. In addition to its canonical role in transcriptional activation, recent studies have demonstrated that *S. cerevisiae* Mediator can interact directly with nucleosomes, and their histone tails. Mutations in Mediator subunits have shown that Mediator and certain chromatin structures mutually impact each other structurally and functionally *in vivo*. We have taken a UV photo cross-linking approach to further delineate the molecular basis of Mediator chromatin interactions and help determine whether the impact of certain Mediator mutants on chromatin is direct. Specifically, by using histone tail peptides substituted with an amino acid analog that is a UV activatible crosslinker, we have identified specific subunits within Mediator that participate in histone tail interactions. Using Mediator purified from mutant yeast strains we have evaluated the impact of these subunits on histone tail binding. This analysis has identified the Med5 subunit of Mediator as a target for histone tail interactions and suggests that the previously observed effect of med5 mutations on telomeric heterochromatin and silencing is direct.

## Introduction

The eukaryotic Mediator complex is a transcriptional co-activator for a wide variety of DNA-bound transcription factors and also serves additional intricate roles in the regulation of transcription [1]. The core of the *S. cerevisiae* complex is composed of 21 polypeptides [2]–[4], which biochemical [5] and structural studies [6] have assigned to structurally distinct modules of the Mediator complex referred to as Tail, Middle and Head. In addition, a separate subset of proteins termed the Cdk8 module is variably associated with the core Mediator subunits [7], [8]. Definitive genomic and proteomic analyses have revealed orthologs for nearly all yeast Mediator subunits in higher eukaryotes [9]–[11]. Parallel biochemical and genetic experiments showed that certain subunits are critical for the activation of specific sets of genes [2], [12]. Transcriptional profiling *in vivo* demonstrated that other Mediator subunits are essential for transcription of virtually all genes in *S. cerevisiae* [13], suggesting the complex was also a general transcription factor. A number of genetic screens and experiments in *S. cerevisiae* have also established an important role for some Mediator subunits in transcriptional repression and silencing [14]–[20]. Our recent work on telomeric silencing [21] and Mediator-chromatin [22] interactions suggests that the mechanism used by Mediator to facilitate repression involves an effect on chromatin.

Genome wide array studies have mapped Mediator occupancy across entire chromosomes in *S. cerevisiae* [23] and *S. pombe* [24]. These studies revealed a uniformly composed core complex upstream of active genes, but unexpectedly also upstream of inactive genes and on the coding regions of some genes. Mediator occupancy was also detected in transcriptionally silent regions of yeast chromosomes, such as telomeres. Recent work has shown that Mediator localizes to telomeres [21] independent of Rap1 and the Sir proteins [25]. Mutations in several Mediator subunits, which result in decreased Mediator occupancy at telomeres, also lead to an increase in H4K16 acetylation, displacement of Sir proteins, and desilencing of telomeric reporter genes [21], [22], [25]. *In vivo* and *in vitro* studies suggest that Mediator does not bind coincidentally with Sir proteins [21]. The occupancy of Mediator near to, but not in, X elements suggest that Mediator may play a critical role in formation of the boundary between heterochromatin and euchromatin at telomeres. How Mediator targeting to telomeres occurs and how it facilitates telomeric silencing are important questions. Our studies of Mediator-chromatin interactions have begun to yield insight into this question.

Consistent with the observation that purified Mediator and mono-nucleosomes directly interact with each other [26], a broad correlation between Mediator occupancy and nucleosome occupancy *in vivo* has been observed [22]. In this same study, it was additionally demonstrated that purified Mediator specifically binds the N-terminal tails of histones H3 and H4. Mediator binding to H4 tail peptides is decreased by the acetylation of lysines in this peptide. Of the most commonly acetylated lysines, the acetylation of H4K16 causes the most significant decrease in affinity of Mediator for the N-terminal tail peptides. These findings were validated by ChIP-chip analysis [22]. Although there is a broad positive correlation between Mediator and nucleosome occupancy *in vivo*, we specifically observed a strong negative correlation between Mediator and nucleosomes acetylated at histone H4 lysine 16. Since deacetylated H4 K16 chromatin is a hallmark of silenced heterochromatin [27], [28], these findings suggested that direct interactions between Mediator and a specialized chromatin structure at telomeres could lead to the targeting of Mediator to heterochromatin and its effect on silencing at these loci.

An outstanding question from our recent studies was whether the same Mediator subunits that impacted telomeric silencing, such as Med5(Nut1)p, also impacted Mediator Histone tail interactions [21]. In this study we have identified several Mediator subunits that interact with histone tails, including Med5(Nut1)p. We have observed that deletion of Med5(Nut1)p leads to a decreased affinity of Mediator for the N-terminal tail of histone H4, suggesting a direct connection between Mediator histone tail binding and silencing.

## Results

### Two Bpa-containing H4 Tail Peptide Derivatives, H4 10 Bpa and H4 22 Bpa, Retain Wild Type Levels of Mediator Binding

**Figure 1:**
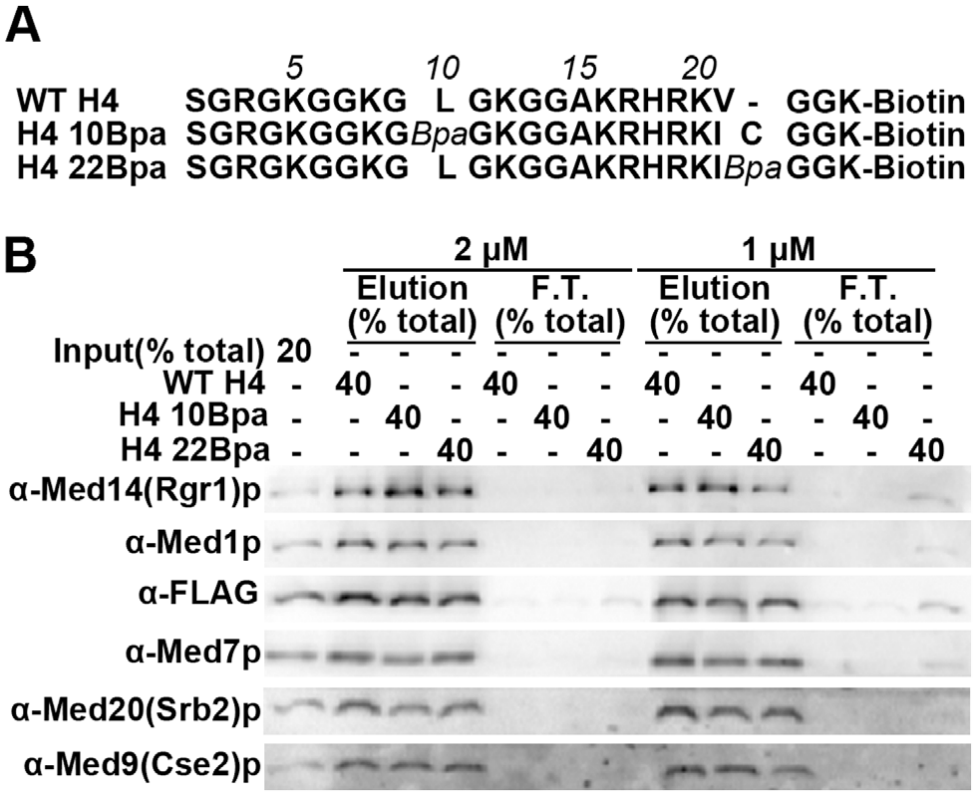
H4 10 Bpa and H4 22 Bpa retain wild type levels of Mediator binding. (A) Sequence alignment of WT H4, H4 10 Bpa and H4 22 Bpa peptide used in binding and cross-linking experiments. (B) Western blot analysis of histone tail binding experiment comparing Mediator binding affinity of WT H4, H4 10 Bpa and H4 22 Bpa. WT Mediator complex (∼3 nM) was mixed with each biotinylated peptide (2 µM or 1 µM) (Input). After incubation with streptavidin beads, Mediator not associated with peptide was in the supernatant and saved as the flow-through fraction (F.T.). Peptide-bound Mediator complex was eluted by boiling the beads in SDS-PAGE loading dye (Elution). The indicated percent of each sample was analyzed by a Western blot in which the specific amount of Mediator was quantified by the specified antibodies against subunits from different structural modules.

We adopted Bpa(benzoyl-phenylalanine)-mediated UV cross-linking as a method to identify the Mediator subunits that are in close proximity to the H4 tail peptide when it is bound to Mediator. Bpa, a photoactivatable derivative of phenylalanine, can be synthetically incorporated into peptides. Upon UV (∼350 nm) irradiation, the activated Bpa group tends to attack C–H bonds, which are geometrically accessible, and form a covalent bond between the Bpa-containing protein or peptide and its binding partner [29]. Two Bpa-incorporated H4 tail probes, H4 10 Bpa and H4 22 Bpa, were synthesized, in which Bpa was substituted for a leucine at position 10 or appended to the C-terminus at position 22 (Fig. 1-A). The concentration of H4 10 Bpa and H4 22 Bpa were able to be normalized to WT H4 peptide by SDS-PAGE and Coomassie blue staining since the peptides had virtually identical sequences (Fig. S1). To test if Bpa-incorporation into the peptides compromised Mediator binding, the affinity of Mediator for H4 10 Bpa, H422 Bpa and WT H4 was compared in the histone tail binding experiment. Consistent with what was observed previously [22], 2 µM of WT H4 peptide was sufficient to deplete Mediator (∼3 nM) complex in the input. H4 10 Bpa and H4 22 Bpa peptides retain the ability to deplete Mediator at 2 µM concentration (Fig. 1-B). Even at a concentration of 1 µM, all three peptides were still capable of pulling down Mediator, indicating that H4 10 Bpa, H4 22 Bpa and WT H4 have comparable affinity for Mediator.

### H4 10 Bpa has Four Specific Cross-linking Targets within Mediator

**Figure 2:**
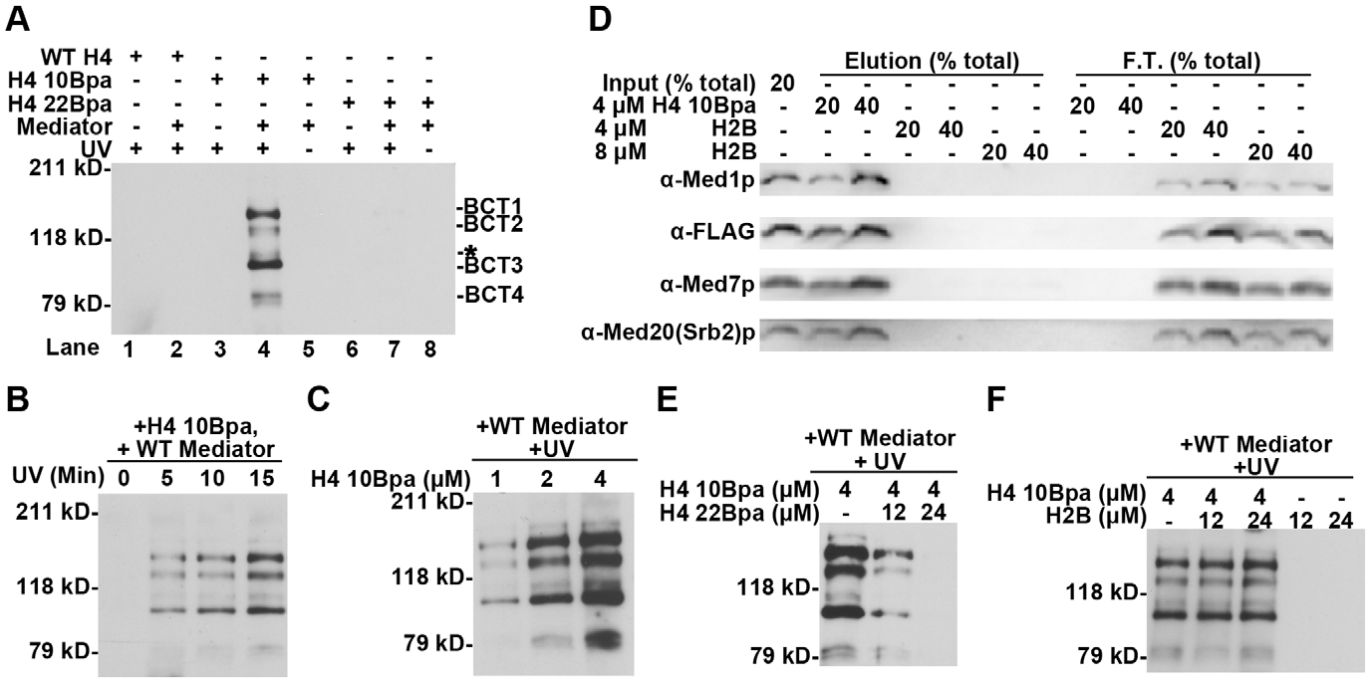
H4 10 Bpa has four specific cross-linking targets within Mediator. (A) SDS-PAGE blot probed with streptavidin poly-HRP to detect biotinylated peptide cross-linked to Mediator subunits. WT H4, H4 10 Bpa and H4 22 Bpa (4 µM) were incubated in the presence or absence of the WT Mediator complex (∼7.5 nM) and exposed to UV for 15 min when indicated. Cross-linking products were resolved on 6% SDS-polyacrylamide gel, transferred to to PVDF, detected by streptavidin poly-HRP, and referred to as BCTs (H4 10 **B**pa **C**ross-linking **T**argets). A weak band with relatively poor reproducibility was asterisked. (B) SDS-PAGE blot probed with streptavidin poly-HRP to detect biotinylated H4 10 Bpa peptide cross-linked to Mediator subunits after different UV exposure times. Identical mixtures, which contain 7.5 nM Mediator complex and 4 µM H4 10 Bpa, were exposed to UV for 0, 5, 10 or 15 min. (C) SDS-PAGE blot probed with streptavidin poly-HRP to detect biotinylated H4 10 Bpa peptide cross-linked to Mediator subunits in reactions with varient H4 10 Bpa concentration. WT Mediator complex (∼7.5 nM) was incubated with 1 µM, 2 µM or 4 µM H4 10 Bpa peptide and exposed to UV irradiation for 15 min. (D) Western blot analysis of histone tail peptide binding experiment comparing Mediator binding affinity for H4 10 Bpa and H2B tail peptide under the identical concentrations to the cross-linking reactions. WT Mediator complex (∼3 nM) was mixed with H4 10 Bpa (4 µM) or synthetic biotinylated histone H2B N’-tail peptide (4 µM or 8 µM) as the inputs. The basic steps and layout of the analysis were as described earlier (Fig. 1). (E) and (F) SDS-PAGE blot probed with streptavidin poly-HRP to detect biotinylated H4 10 Bpa peptide cross-linked to Mediator subunits, after H4 22 Bpa (E) or H2B tail peptide (F) was added at the indicated concentration.

Our strategy to identify Mediator subunits that are on, or proximate to, the H4 tail binding interface was to covalently label these subunits with the Bpa-containing probes. Cross-linked target proteins were detectable by streptavidin poly-HRP, since the probes were biotinylated at their C-termini. Two strong and two weak biotinylated bands on SDS-PAGE were observed when the H4 10 Bpa and Mediator mixture was exposed to UV irradiation (Fig. 2-A Lane 4). Using non-Bpa-containing WT H4 tail peptide as the probe (Lane 1, 2), omitting UV irradiation (Lane 5), or omitting Mediator in the reaction (Lane 3) all resulted in the absence of these signals on the blot (Fig. 2-A). These data demonstrate that the observed pattern in Lane 4 specifically results from a Bpa-mediated covalent cross-link between Mediator and H4 10 Bpa probe. By analyzing H4 10 Bpa cross-linking products on a 10% SDS gel (Fig. S2), we did not observe any other significant cross-linking signals in the lower molecular weight range. The four bands with high intensity and good reproducibility in the cross-linking pattern were designated as BCT1 (H4 10 **B**pa **C**ross-linking **T**arget 1), BCT2, BCT3 and BCT4 (Figure 2-A). The BCT1 and BCT3 signals were significantly and reproducibly stronger than BCT2 and BCT4, suggesting that BCT1 and BCT3 may be the primary targets of H4 tail binding. Distinct from H4 10 Bpa, H4 22 Bpa failed to generate detectable cross-linking signals under the same conditions (Fig. 2-A, Lane 7). The H4 22 Bpa cross-linking samples analyzed by 10% SDS-PAGE also showed no detectable signal in the lower molecular weight range (data not shown). It is likely that position 22 is too distant from the H4 binding site for the Bpa group to achieve efficient cross-linking. Additional experiments were used to further address the specificity of the H4 10 Bpa cross-linking result. First, the UV exposure time and H4 10 Bpa concentration were reduced. Fig. 2-B and 2-C show that shortening UV irradiation or decreasing H4 10 Bpa concentration only led to general weakening of the cross-linking signals, without significantly changing the cross-linking pattern. Second, since the standard concentration of H4 10 Bpa in cross-linking reactions was 4 µM, which was higher than the concentration typically used for binding assays (1 µM or 2 µM), we wanted to rule out non-specific binding resulting from a higher peptide concentration. As shown in Fig. 2-D, 4 µM H4 10 Bpa depleted Mediator in the input, while H2B tail peptide (the non-specific binding control [22]) was not able to pull down detectable amounts of the complex, at identical or even double the concentration. The interaction between Mediator complex and histone tail peptides retains its specificity under the cross-linking conditions. Third, H4 22 Bpa, which bound Mediator (Fig. 1-B), but did not cross-link to the complex (Fig. 2-A Lane 7), was able to compete with H4 10 Bpa in cross-linking experiments and attenuate the BCT1-4 signals (Fig. 2-E). This attenuation was specific as similar amounts of H2B peptide were not able to recapitulate the effect (Fig. 2-F). We conclude that the H4 10 Bpa cross-linking pattern relies on the specific interaction between Mediator complex and H4 10 Bpa peptide.

### Med5(Nut1)p, Med14(Rgr1)p, Med17(Srb4)p and Med1p are H4 10 Bpa Cross-linking Targets

**Figure 3:**
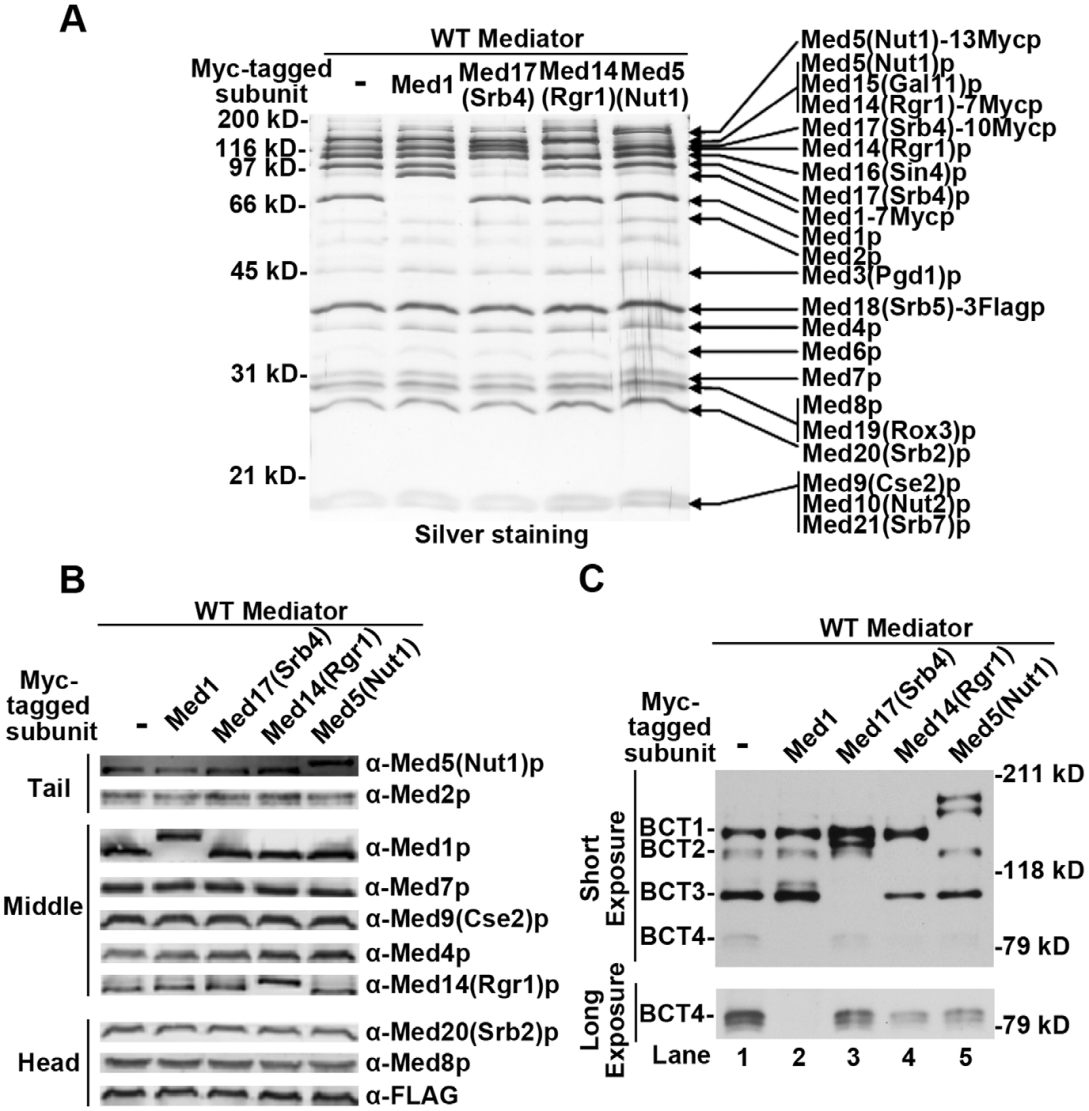
Med5(Nut1)p, Med14(Rgr1)p, Med17(Srb4)p and Med1p are H4 10 Bpa cross-linking targets. Silver staining (A) and immunoblotting analysis (B) comparing the composition of affinity-purified *MYC*-tagged and non-*MYC*-tagged WT Mediator complexes after 10% SDS-PAGE. (C) A comparison of the cross-linking patterns of *MYC*-tagged Mediator complexes with the WT pattern using an SDS-PAGE blot probed with streptavidin poly-HRP to detect biotinylated H4 10 Bpa peptide cross-linked to Mediator subunits. Each indicated Mediator species (∼7.5 nM) was incubated with H4 10 Bpa (4 µM). A ‘Long Exposure’ of the 79 kD region on the SDS-PAGE blot probed with streptavidin poly-HRP is shown for a better view of the weak BCT4 signal in each sample.

**Figure 4:**
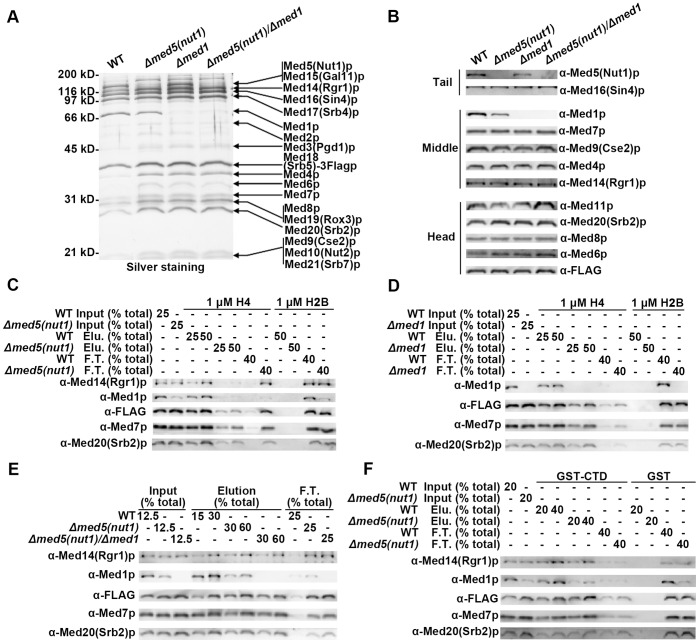
Med5(Nut1)p is important for Mediator-H4 interaction, while Med1p is not. Silver staining (A) and immunoblotting analysis (B) comparing the composition of affinity-purified *Δmed5(nut1), Δmed1*, and *Δmed5(nut1)/Δmed1* and WT Mediator complexes after 10% SDS-PAGE. The amount of each complex in the individual lanes was normalized by adjusting the load such that an equal signal from the α-flag and α-Med7 antibodies was present. (C) Western blot analysis of histone tail binding experiment comparing the H4 tail binding affinity of WT and *Δmed5(nut1)* Mediator complexes. An equal concentration (∼3 nM) of either WT or *Δmed5(nut1)* Mediator complex was mixed with WT H4 peptide (1 µM) and with H2B peptide (1 µM) as the inputs. The basic steps and layout of the analysis were as described earlier (Fig. 1). (D) Western blot analysis of histone tail binding experiment comparing the H4 tail binding affinity between WT and *Δmed1* Mediator complexes. An equal concentration (∼3 nM) of WT or *Δmed1* Mediator complex was mixed with WT H4 peptide (1 µM) and with H2B peptide (1 µM) as the inputs. (E) Western blot analysis of histone tail binding experiment comparing the H4 tail binding affinity of WT, *Δmed5(nut1)* and *Δmed5(nut1)/Δmed1* Mediator complexes. An equal concentration (∼6 nM) of WT, *Δmed5(nut1)* or *Δmed5(nut1)/Δmed1* Mediator complex was mixed with WT H4 peptide (1.4 µM) as the inputs. (F) Western blot analysis of GST-CTD pull down experiment comparing the GST-CTD binding affinity for WT and *Δmed5(nut1)* Mediator complexes. An equal concentration (∼15 nM) of WT or *Δmed5(nut1)* Mediator complex (Input) was incubated with glutathione beads, which were pre-loaded with equal amounts of GST-CTD or GST. After incubation, the supernatant was saved as Flow-though (F.T.). Bound protein was eluted from the beads by boiling them in SDS-PAGE loading dye (Elution). An indicated percent of each fraction was analyzed by immunoblotting using the specified antibodies against Mediator subunits from different structural modules.

**Figure 5:**
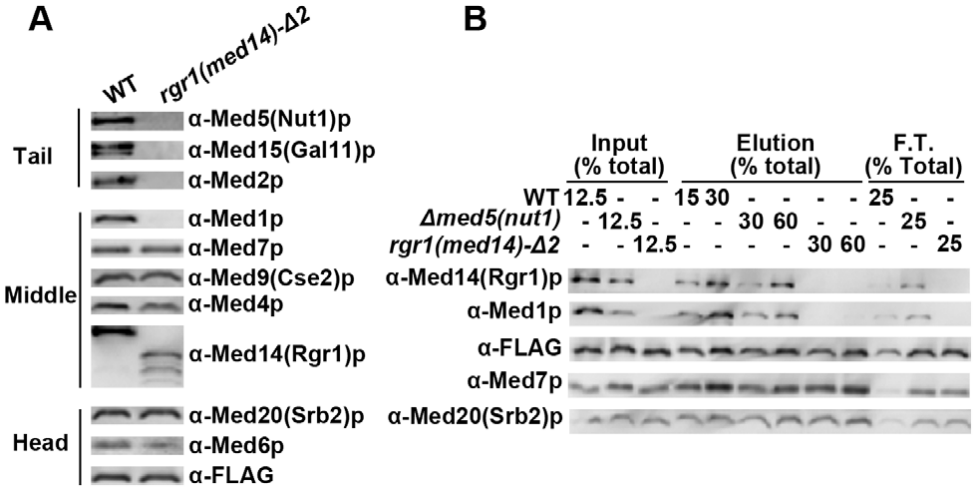
The C-terminus of Med14(Rgr1)p does not directly contribute to H4-Mediator interaction. (A) Immunoblotting analysis comparing the composition of affinity-purified *rgr1(Med14)-Δ2* and WT Mediator complexes after 10% SDS-PAGE. (B) Western blot analysis of histone tail binding experiment comparing the H4 tail binding affinity of WT, *Δmed5(nut1)* and *rgr1(Med14)-Δ2* Mediator complexes. An equal concentration (∼6 nM) of WT, *Δmed5(nut1)* or *rgr1(Med14)-Δ2* Mediator complex was mixed with WT H4 peptide (1.4 µM) as the inputs. The basic steps and layout of the analysis were as described earlier (Fig. 1).

We used the approximate molecular weight of each BCT to make a preliminary identification of the subunits of Mediator that cross-linked to H4 10 Bpa. Referring to the silver staining pattern of the WT Mediator complex, we assigned BCT2, BCT3 and BCT4 as the H4 10 Bpa cross-linked form of Med14(Rgr1)p, Med17(Srb4)p and Med1p respectively with high confidence. The two largest subunits of Mediator, Med15(Gal11)p and Med5(Nut1)p, have similar molecular weights and co-migrate on SDS-PAGE. Therefore, BCT1 could be the cross-linking products from either Med15(Gal11)p or Med5(Nut1) or both. We designed an epitope tagging strategy to confirm the preliminary assignments. The principle of this strategy was that if a BCT signal was correctly assigned, increasing the molecular weight of the target Mediator subunit by tandem-Myc-tagging would alter the cross-linking pattern by shifting the corresponding BCT signal to a higher molecular weight. For this purpose, *MED14(RGR1), MED17(SRB4)* and *MED1* were each individually C’-*MYC*-tagged in the *MED18(SRB5)-3XFLAG* background. These strains enabled the affinity purification of each Mediator complex. We also *MYC*-tagged *MED5(NUT1)* as an attempt to clarify the subunit(s) represented by BCT1. The Myc-tagged Mediator complexes were purified and compared with non-tagged WT Mediator complex by silver staining (Fig. 3-A) and immunoblotting (Fig. 3-B). Both methods validated the successful Myc-tagging and the integrity of these Mediator complexes during purification. The Myc-tagged Mediator complexes were found to bind to WT H4 tail (data not shown), and H4 10 Bpa (Fig. S3), with equal affinity to non-Myc-tagged WT Mediator. The cross-linking pattern of the Med17(Srb4)-10Myc Mediator complex (Fig. 3-C Lane 3) shows that the original BCT3 band was absent and that a new signal could be clearly observed between BCT1 and BCT2. This result indicates BCT3 was correctly assigned and represents the H4 10 Bpa labeled form of Med17(Srb4)p. Similarly, a shift of BCT1 was found in the cross-linking pattern of the Med5(Nut1)-13Myc Mediator complex (Fig. 3-C Lane 5), indicating Med5(Nut1)p is the only cross-linking target which generates BCT1 signal. It is unclear why the H4 10 Bpa labeled form of Med5(Nut1)-13Myc protein appeared as a doublet. In the cross-linking patterns of the Med14(Rgr1)-7Myc and Med1-7Myc Mediator complexes (Fig. 3-C Lane 4 and Lane 2), we observed the evident elimination of BCT2 and BCT4 respectively. It was not readily apparent where these two weaker BCT signals shifted. Given the distinct molecular weight of Med14(Rgr1)p and Med1p, the chances are low that assignment of BCT2 and BCT4 is incorrect. One interpretation is that Med1p and Rgr1p may act as H4 10 Bpa weak transient tethering sites, or just happen to be spatially proximal to the direct binding sites and therefore get cross-linked by H4 10 Bpa. It is possible that this weak interaction or proximity can be disrupted by Myc-tagging, thus resulting in Myc-tagged Med1p or Med14(Rgr1)p no longer being H4 10 Bpa cross-linking targets. The idea that Med1p and Med14(Rgr1)p have weak H4 tail interactions is supported by our H4 binding assays that show mutations in Med1p and Med14(Rgr1)p have little direct impact on the affinity of Mediator for H4 tail peptide (See Figs. 4 and 5). Another explanation for the inability to detect the H4 10 Bpa cross-linked form of Med14(Rgr1)-7Mycp could be its co-migration with the cross-linked form of WT Med5(Nut1)p. Additional data that further support our assignment of the BCT1-4 are discussed later in the results section. In total, the evidence convincingly supports the identification Med5(Nut1)p and Med17(Srb4)p as strong H4 10 Bpa cross-linking targets, and Med14(Rgr1)p and Med1p as H4 10 Bpa weak cross-linking targets.

### The *Δmed5(nut1)* Mediator Complex has Compromised Affinity for H4 Tail Peptide

To study the role of H4 10 Bpa cross-linking targets in H4 tail binding, we undertook the purification and characterization of Mediator from strains with mutations in the cross-linking target. We started by purifying the *Δmed5(nut1)* Mediator. As an alternative to the previous conventional *Δmed5(nut1)* Mediator purification [30], we constructed a *Δmed5(nut1)*, *MED18(SRB5)-3FLAG* yeast strain for affinity purification. We purified *Δmed5(nut1)* Mediator and compared its composition with WT Mediator complex by silver staining and immunoblotting (Fig. 4-A and 4-B). Both methods clearly showed that Med5(Nut1)p is absent in the mutant Mediator, while the structural integrity of the remaining complex is intact. We also found that Med1p, another H4 10 Bpa cross-linking target, is substoichiometric in the purified *Δmed5(nut1)* Mediator, suggesting that Med5(Nut1)p may affect the assembly or stability of Med1p in the complex. This result also supports the suggested spatial proximity between the two proteins within the complex [3]. Next, we assayed the H4 tail binding affinity of the *Δmed5(nut1)* Mediator. Compared with the WT Mediator complex, significantly less *Δmed5(nut1)* Mediator was pulled-down by H4 tail peptide, and much of the mutant complex remained in the flow-through fraction (Fig. 4-C). This defect is unlikely to be a result of a sub-population of “inactive” Mediator. Equimolar amounts of purified wild type and *Δmed5(nut1)* Mediator have equivalent binding affinity for CTD (C-terminal domain of RNA polymerase II), a *bona fide* binding partner of Mediator [31], in a GST-CTD pull-down experiment (Fig. 4-F). Combined, the above results indicate that the *Δmed5(nut1)* Mediator, relative to wild type, has a decreased affinity specifically for H4 tail peptide.

### Missing Med1p in Mediator Complex does not Compromise H4 Tail Binding

From the above result, it is unclear if the impaired H4 tail binding affinity in the *Δmed5(nut1)* Mediator is caused by the absence of Med5(Nut1)p, substoichiometric amounts of Med1p, or both. To clarify this question, first we purified the *Δmed1* Mediator complex. The silver staining pattern (Fig. 4-A) and immunoblotting analysis (Fig. 4-B) showed that other than the absence of Med1, no Mediator subunits are detectably substoichiometric in the *Δmed1* Mediator. When characterized in the H4 tail binding assay, *Δmed1* Mediator and WT Mediator were found to have comparable affinity for H4 tail peptide (Fig. 4-D). A very mild decrease in the affinity of the *Δmed1* Mediator was sometimes observed, but was not reproducible and could have resulted from small decreases in the amount of Med5p. The result indicates that Med1p does not impact the Mediator-H4 tail interaction, and that the decreased H4 tail binding affinity of *Δmed5(nut1)* Mediator does not result from the substoichiometric amounts of Med1p in the mutant complex. To validate this second conclusion, we purified Mediator from the *Δmed5(nut1)/Δmed1* strain. Aside from missing Med5(Nut1)p and Med1p, no significant compositional change was detected in *Δmed5(nut1)/Δmed1* Mediator (Fig. 4-A and 4-B). The *Δmed5(nut1)/Δmed1* Mediator did not show any further decrease in H4 tail binding affinity when compared with the *Δmed5(nut1)* Mediator (Fig. 4-E). In the experiment in Fig. 4-E the concentration of both Mediator and peptide was increased, compared to the experiment in Fig. 4-C, in order to allow for the sensitivity to evaluate any further decreases in affinity caused by the absence/mutation of subunits in addition to Med5(Nut1)p. The absence of Med1p, in the context of a Med5(Nut1)p deletion, has no further impact on Mediator-H4 tail binding.

### The C-terminus of Med14(Rgr1) does not Directly Contribute to the H4 Tail Binding Affinity of Mediator

Med14(Rgr1)p, unlike Med1p and Med5(Nut1)p, is a H4 10 BPA cross-linking target encoded by an essential gene. Therefore, we used a biochemically well-characterized C-terminal truncation mutant, *med14(rgr1)-Δ2* [32], [33],to study the role of Med14(Rgr1)p in Mediator and H4 tail interactions. Flag affinity purification was used to isolate the *med14(rgr1)-Δ2* Mediator and the composition was compared to wild type Mediator by immunoblotting analysis (Fig. 5-A). Consistent with previous studies, *med14(rgr1)-Δ2* truncation leads to the complete dissociation of the tail module [32], [33]. We also did not detect the presence of Med1p, which is a reported component of *med14(rgr1)-Δ2* Mediator purified by conventional methods [32], [33]. Since Med5(Nut1)p was absent from the *med14(rgr1)-Δ2* Mediator, we predicted the *med14(rgr1)-Δ2* mutant complex should have a decreased H4 tail binding affinity. The decrease in affinity of the *med14(rgr1)-Δ2* Mediator for H4 tail is comparable to the *Δmed5(nut1)* mutant complex (Fig. 5-B). This finding suggests that the subunits absent in the *med14(rgr1)-Δ2* Mediator (the C-terminal part of Med14(Rgr1)p, Med16(Sin4)p Med15(Gal11)p, Med2p, Med3(Pgd1)p, and Med1p) do not make any direct contribution to Mediator-H4 tail binding beyond the absence of Med5(Nut1)p.

### Non-crosslinked Mediator Subunits, Med16(Sin4)p and Med9(Cse2)p, do not Directly Influence H4 Tail Binding

**Figure 6:**
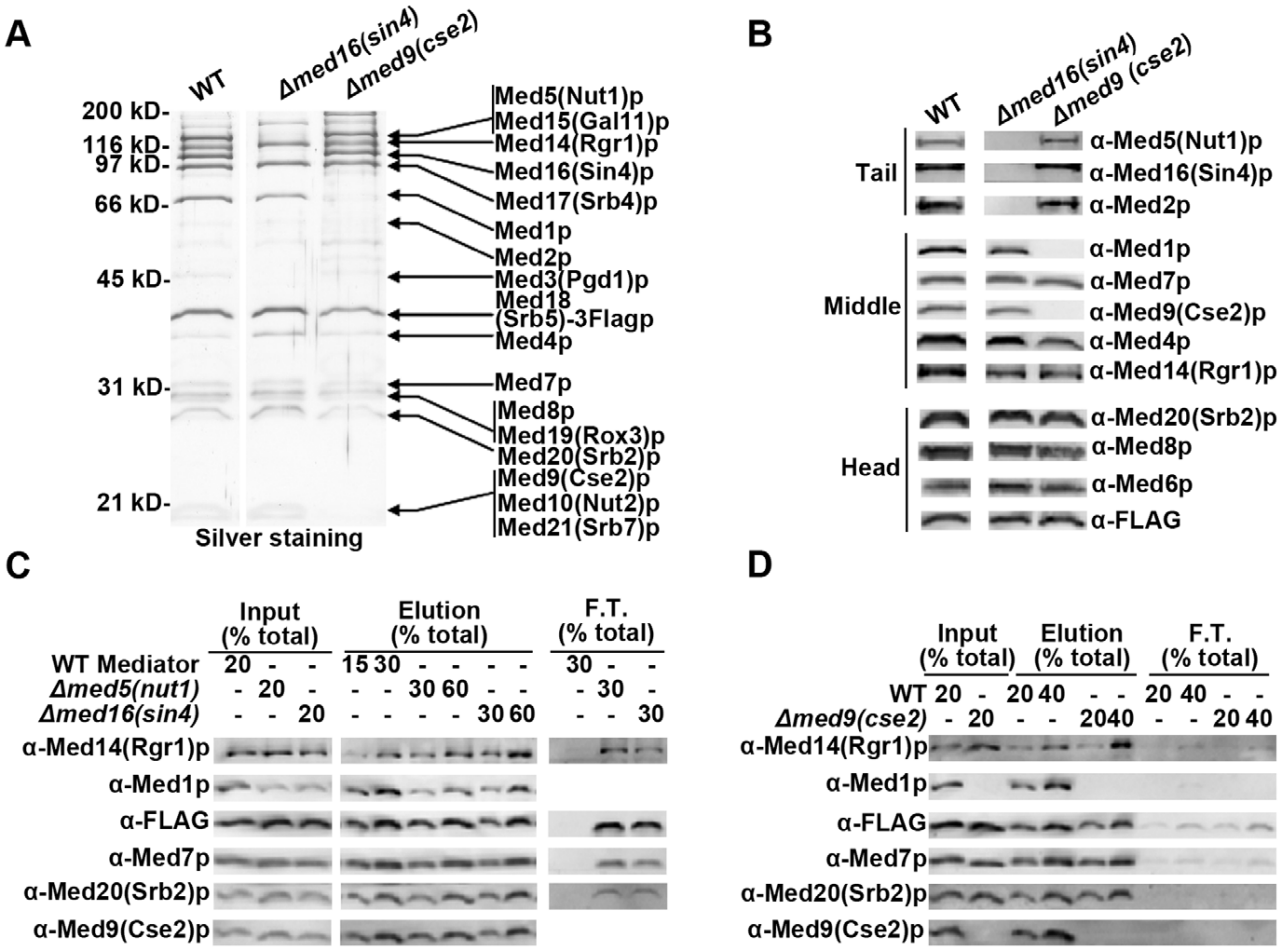
Med16(Sin4)p and Med9(Cse2)p do not directly influence H4 tail binding. Silver staining (A) and immunoblotting analysis (B) comparing the composition of affinity-purified *Δmed16(sin4)*, *Δmed9(cse2)* and WT Mediator complexes after 10% SDS-PAGE. (C) Western blot analysis of histone tail binding experiment comparing the H4 tail binding affinity of WT, *Δmed5(nut1)*, and *Δmed16(sin4)* Mediator complexes. An equal concentration (∼4.7 nM) of WT, *Δmed5(nut1)*, or *Δmed16(sin4)* Mediator complex was mixed with WT H4 peptide (1.4 µM) as the inputs. The basic steps and layout of the analysis were as described earlier (Fig. 1). (D) Western analysis of histone tail binding experiment comparing the H4 tail binding affinity of WT and *Δmed9(cse2)* Mediator complexes. An equal concentration (∼3 nM) of WT or *Δmed9(cse2)* Mediator complex was mixed with WT H4 peptide (1 µM) as the inputs.

To determine whether other Mediator subunits implicated in chromatin related effects, other than those identified as direct cross-linking targets, could indirectly influence H4 binding we tested Mediator purified from a *Δmed16(sin4)* strain and a *Δmed9(cse2) strain.* Med16(Sin4)p mutations are accompanied by gross alterations in chromatin structure *in vivo*, and lead to the de-repression of a subset of genes, potentially by an epigenetic mechanism [14], [34], [35]. Med9(Cse2)p, a middle module subunit, has also been shown to be important for transcriptional repression [17]. We purified the *Δmed16(sin4)* and *Δmed9(cse2)* Mediator complexes using Flag-tagged strains. Med15(Gal11)p, Med2p, Med3(Pgd1)p and Med5(Nut1)p have been shown to be lost from the conventionally purified *Δmed16(sin4)* Mediator [30], [36]. By silver staining (Fig. 6-A) and immunoblotting analysis (Fig. 6-B), we confirmed the complete dissociation of the tail module and the integrity of the middle and head modules in purified *Δmed16(sin4)* Mediator [30], [36]. Consistent with the absence of Med5(Nut1)p in the *Δmed16(sin4)* Mediator, we also noted substoichiometric amounts of Med1p. Conventionally purified *Δmed9(cse2)* Mediator has been shown to lack Med1p and have substoichiometric amounts of Med4p [37]. *Δmed9(cse2)* Mediator complex purified by affinity approach recapitulated the above compositional characteristics, as shown by silver staining (Fig. 6-A) and immunoblotting (Fig. 6-B). As shown in Fig. 6-C, the absence of Med16(Sin4)p and other Tail module subunits does not result in further compromised H4 tail binding affinity when compared with the *Δmed5(nut1)* Mediator. Combined with the H4 tail binding data of the *rgr1(med14)-Δ2* Mediator (Fig. 5), this result further reinforced the conclusion that no Mediator tail module subunits influence H4 tail binding through non-Med5(Nut1)p dependent mechanisms. Furthermore, purified *Δmed9(cse2)* Mediator bound to H4 tail peptide as tight as WT mediator complex (Fig. 6-D), indicating Med9(Cse2)p and Med4p do not play important roles in H4 tail binding.

### H4 10 Bpa Cross-linking Patterns of the Mutant Mediator Complexes Further Define Mediator-H4 Tail Interactions

**Figure 7:**
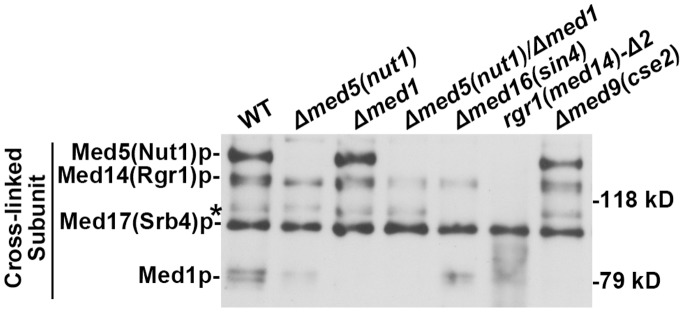
H4 10 Bpa cross-linking patterns of the mutant Mediator complexes. SDS-PAGE blot probed with streptavidin poly-HRP to detect and compare the pattern of biotinylated peptide cross-linked to Mediator subunits in WT Mediator and the mutant Mediator complexes. Equimolar amounts (∼7.5 nM) of each indicated Mediator complex were incubated with H4 10 Bpa (4 µM) and exposed to UV for 15 min. Asterisk refers to the same weak signal as in Fig. 2-A.

Using the H4 10 Bpa probe and the above-characterized mutant Mediator complexes, we were able to generate the H4 10 Bpa cross-linking pattern for each mutant Mediator (Fig. 7). Several conclusions can be derived from this data. First, the results further confirm Med5(Nut1)p, Med14(Rgr1)p and Med1p as H4 10 Bpa cross-linking targets. The Med5(Nut1)p cross-linking signal (BCT1) was absent from the cross-linking patterns of the *Δmed5(nut1), Δmed5(nut1)/Δmed1, Δmed16(sin4)* and *rgr1(Med14)-Δ2* Mediator complexes, none of which contained detectable levels of Med5(Nut1)p. Similarly, the Med1p cross-linking product (BCT4) was absent in the *Δmed1, Δmed5(nut1)/Δmed1, rgr1(Med14)-Δ2* and *Δmed9(cse2)* cross-linking patterns. Additionally, the full length Med14(Rgr1)p cross-linking signal (BCT2) was not present in the cross-linking pattern of the *rgr1(Med14)-Δ2* Mediator complex. Second, the weak cross-linking signal with poor reproducibility, which is marked in Fig. 2-A, S2 and Fig. 7, is likely to represent occasionally cross-linked Med16(Sin4)p since this band was completely abolished in the cross-linking patterns of the *Δmed16(sin4)* and *rgr1(Med14)-Δ2* Mediator complexes. Third, there is no readily identified signal indicating that the truncated form of Med14(Rgr1)p was cross-linked to H4 tail in the *rgr1(Med14)-Δ2* Mediator complex cross-linking pattern. The potential overlap of the truncated protein signal with Med17(Srb4) as well as split signal of the rgr1(Med14)-Δ2p in Western blots (Fig. 5-A), however, make difficult to rule this out. This result suggests that H4 10 Bpa cross-linking targets the C-terminal part of Med14(Rgr1)p. Med17(Srb4)p produces the only cross-linking signal in the *rgr1(Med14)-Δ2* Mediator pattern. An interaction between Med17(Srb4)p and the H4 tail is likely to be responsible for the residual H4 tail binding affinity of this mutant complex. Finally, we did not observe any interdependency among the cross-linking signals. Hence, even though Med16(Rgr1)p and Med1p may be proximal to Med5(Nut1)p, the H4 tail cross-linking almost certainly results from a weak interaction with these subunits rather than their proximity to the tighter interaction with Med5(Nut1)p.

## Discussion

Our earlier work showed that Med5p(Nut1)p was required for association of Mediator with the specialized chromatin structure at yeast telomeres and the maintenance of silenced heterochromatin *in vivo* [21]. Another branch of our work showed that Mediator can interact with nucleosomes through the histone tails and that a modification associated with silenced heterochromatin, H4 K16deAc, was important for this interaction [22]. It was an open question whether the effect of Med5(Nut1)p on silenced heterochromatin resulted from a direct effect on histone tail interactions. In this study we have taken an unbiased approach to identifying subunits within Mediator that interact directly with histone H4 N-terminal tail. Our analysis identified Med5(Nut1)p and, by process of elimination, Med17(Srb4)p as the sites on Mediator that provide the primary affinity for H4 tail. The identification of Med5(Nut1)p as a subunit that contributes to the affinity of Mediator for H4 further elucidates the previously observed effect of Med5(Nut1)p on telomeric silencing. This result supports the idea that the observed effects of med5(nut1) deletion on telomeric silencing *in vivo* directly result from the absence of a Med5(Nut1)p-H4 tail interaction. A second report on *S. cerevisiae* Mediator concluded that mutations in several tail module subunits, but not Med5(Nut1)p, lead to the loss of telomeric silencing [25]. Although the origin of the different results is still unclear, it may be related to the different locus of the URA3 marker gene inserted in telomere VIIL used in the above study that lacks the subtelomeric X and Y elements [25].

Med17(Srb4)p is encoded by an essential gene and is considered a key component of the structural core of Mediator [38]–[40]. Hence, it will be difficult to identify a potential contribution of the H4 interaction with Med17(Srb4)p to telomeric silencing and chromatin interactions *in vivo* until a specific binding interface is identified that can be subject to point mutations or small deletions. However, both the strong cross-linking signal, and experiments which show that a Med17(Srb4)p containing Mediator head module, purified under mildly denaturing conditions, binds H4 tail with equal affinity to the *Δmed5(nut1)* Mediator (data not shown) support the idea that Med17(Srb4)p is the second important H4 tail binding site in Mediator.

The next step in this line of inquiry will be to determine specific domains, and even amino acids, within Med5(Nut1)p and Med17(Srb4)p that are required for histone tail binding. Both Med5(Nut1)p and Med17(Srb4)p are relatively large proteins and have many potential sites for interaction with the histone tails. It has previously been noted that residues 1–243 of Med5(Nut1)p contain all four motifs characteristic of the GCN5-related N-acetyltransferase (GNAT) superfamily, with similar spacings of the motifs to those in other family members [26]. Med5(Nut1)p has a weak histone acetyltransferase activity [26] and it is possible that this motif now serves to bind H4 tail peptide. Structural studies of GNAT superfamily member tGCN5 have shown that this motif can help serve as a binding site for both histone H3 and histone H4 N-terminal tails [41]. The identification of both Med5(Nut1)p and Med17(Srb4)p as H4 binding partners suggests that perhaps they might share a common motif used in binding. Although Med17(Srb4)p is not a GNAT superfamily member, a direct pairwise alignment of Med5(Nut1)p and Med17(Srb4)p searching for local similarities (EMBOSS Matcher, EMBL-EBI) between the two proteins revealed an ∼40 amino acid stretch that has high similarity between the two (Med5(Nut1)p a.a. 976–1016, Med17(Srb4)p a.a. 145–186). Intriguingly this stretch has several highly conserved aspartic acids and glutamic acids that might be used to interact with a highly positively charged substrate, such as a histone tail. This region, in Med5(Nut1)p, is not part of the GNAT motifs and is unstructured in the crystal structure of Med17(Srb4)p within the Mediator head module [40]. Coupling mass spectrometry to our photo cross-linking approach and/or systematic deletion of candidate regions in Med5(Nut1)p and Med17(Srb4)p for H4 tail binding will be necessary to design mutations that will allow us to more precisely test the effect of these interactions *in vivo*.

## Materials and Methods

### Yeast Strains Construction

Med18(Srb5)p Flag-tagged strains with *MYC*-tagged Mediator subunits (yZL1, yZL2, yZL3 and yZL4) were constructed by individually targeting each gene in SHY349 [42] with the corresponding PCR product generated from pFA6a-13myc-His3MX6 [43]. To generate yeast strains for purifying mutant Mediator complexes, *MED18(SRB5)-3FLAG-NAT^R^* cassette, which was amplified from yLM40 [44], was used to tag *MED18(SRB5)* in the *Δmed1* (strain #15489), *Δmed9(cse2)* (strain #15385) and *Δmed5(nut1)* (strain #14518) strain from the Saccharomyces Genome Deletion Project Libarary [45] to create yLM79, yZL15 and yLM74 respectively. This cassette was also used for the same purpose in DY2694 (r*gr1-Δ2(med14)::LEU2*) and DY1876 (*Δmed16(sin4)::TRP1*) to generate yZL14 and yLM61 respectively. The *MED1* ORF in yLM74 was deleted by the *HIS3* marker amplified from pFA6a-His3MX6 [44] to generate yZL13. The correct integration of each targeting DNA fragment was confirmed by PCR and the success of the epitope tagging steps were further verified by immunoblotting analysis. The complete genotypes of all strains used in this study are listed in Table S1.

### Affinity Purification of Mediator Complex

In addition to individual genetic modifications, all the yeast strains used for Mediator complex purification in this study had a triple FLAG tag at the C-terminus of MED18(SRB5) gene. Affinity purification of each Mediator complex was performed as previously described [44] with the following modifications. After the salt concentration adjustment, crude cell lysate was first applied on Bio-Rex 70 resin and Mediator eluted as previously described [46]. Mediator containing Bio-Rex fractions eluted at 650 mM KOAc were added to anti-FLAG M2 agarose (Sigma). Mediator complex products, which were eluted by 3XFlag peptide, were further purified by size exclusion chromatography (Superose 6 10/300 GL GE) in 25 mM HEPES KOH (pH 7.6), 5% glycerol, 0.01% NP-40, 300 mM KOAc. Fractions containing intact Mediator were pooled and re-concentrated by anti-FLAG agarose.

### Histone Tail Peptide Binding Experiments

Histone tail binding experiments were performed as previously described [22], except that the total reaction volume was reduced to 50 µl. After incubation with streptavidin beads, the supernatant was collected as flow-through fraction and TCA precipitated for SDS-PAGE analysis. The input peptide concentration was varied as described in the figure captions.

### UV Cross-linking

The H4 10 BPA peptide (SGRGKGGKG(BPA)GKGGAKRHKICGGK-biotin) and the H4 22 BPA (SGRGKGGKGLGKGGAKRHKI(BPA)GGK-biotin) peptide were synthesized at the Tufts University Core Facility. UV cross-linking was conducted in 20 µl F300 buffer [25 mM HEPES KOH (pH 7.6), 10% glycerol, 0.01% NP-40, 300 mM KOAc] containing 4 µM H4 10 BPA (or other H4 tail peptide) and 7.5 nM purified Mediator. If a competitor peptide was present in the experiment, it was added at this stage. The mixture was incubated for 4 h and then exposed to 5 UV tubes (8-watt each, 365 nm) at a distance of 10 cm. Irradiation time was 15 min if not otherwise specified. Cross-linking products were resolved by SDS-PAGE and blotted by Streptavidin Poly-HRP (Thermo), which was 1∶15,000 diluted in TBST+2.5%BSA.

### GST-CTD Pull-down Experiments

10 µg purified GST-CTD [31] or GST was bound to 5 µl of Glutathione MagBeads (Genscript). Unbound proteins were removed by washing the beads in F300 buffer containing 0.5 mM DTT. 10 µl of wild-type or *Δmed5(nut1)* Mediator (∼15 nM) was added to the beads and incubated for 2 h. The supernatant after incubation was saved as the flow-through fraction representing the unbound Mediator. Bound Mediator complex was eluted by boiling the beads in SDS-PAGE loading dye.

### Antibodies

All Western blots were developed using a AP(alkaline phosphatase)-conjugated secondary antibody and ECF (GE Healthcare) reagent. The antibodies used for detecting invidual Mediator subunits were as previously described [44].

## Supporting Information

Figure S1 **Normalization of peptide concentration.** To normalize the amounts of peptide added to binding and cross-linking reactions, WT H4, H4 10 Bpa and H4 22 Bpa synthetic peptide were diluted in SDS-PAGE loading dye and resolved by Tricine-SDS-PAGE. Concentration of the peptides was calibrated by Coomassie blue staining signals.
(TIF)

Figure S2 **H4 10** **Bpa cross-linking pattern resolved by 10% SDS-PAGE.** SDS-PAGE blot probed with streptavidin poly-HRP to detect biotinylated peptide cross-linked to Mediator subunits resolved on 10% SDS-polyacrylamide. The high molecular weight signals are labelled as previously described (Fig. 2-A).
(TIF)

Figure S3 ***Myc*** **-tagging does not interfere with the binding between Mediator complex and H4 10** **Bpa peptide.** Western blot analysis of a histone tail binding experiment in which each indicated Mediator species (∼3 nM) was mixed with H4 10 Bpa (4 µM) as the input. The basic steps and layout of the analysis were as described earlier (Fig. 1).
(TIF)

Table S1 **Yeast strains used in this study.**
(DOCX)
