# Supplementary figures and images for: Med5(Nut1) and Med17(Srb4) Are Direct Targets of Mediator Histone H4 Tail Interactions
Source: PLoS One. 2012 Jun 5;7(6):e38416. doi: 10.1371/journal.pone.0038416 (PMC3367926; doi:10.1371/journal.pone.0038416)

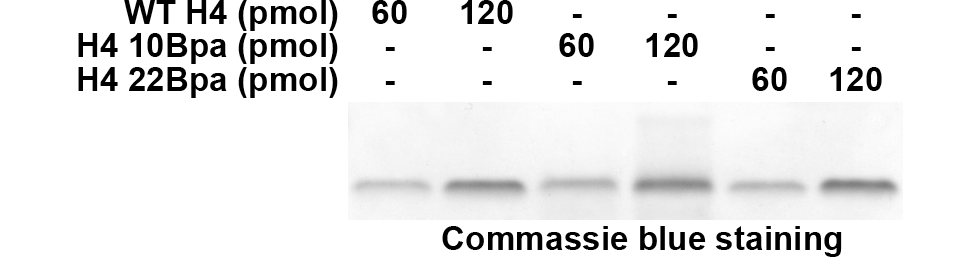

Supplement: Figure S1 — Normalization of peptide concentration. To normalize the amounts of peptide added to binding and cross-linking reactions, WT H4, H4 10 Bpa and H4 22 Bpa synthetic peptide were diluted in SDS-PAGE loading dye and resolved by Tricine-SDS-PAGE. Concentration of the peptides was calibrated by Coomassie blue staining signals. (TIF) [file pone.0038416.s001.tif]

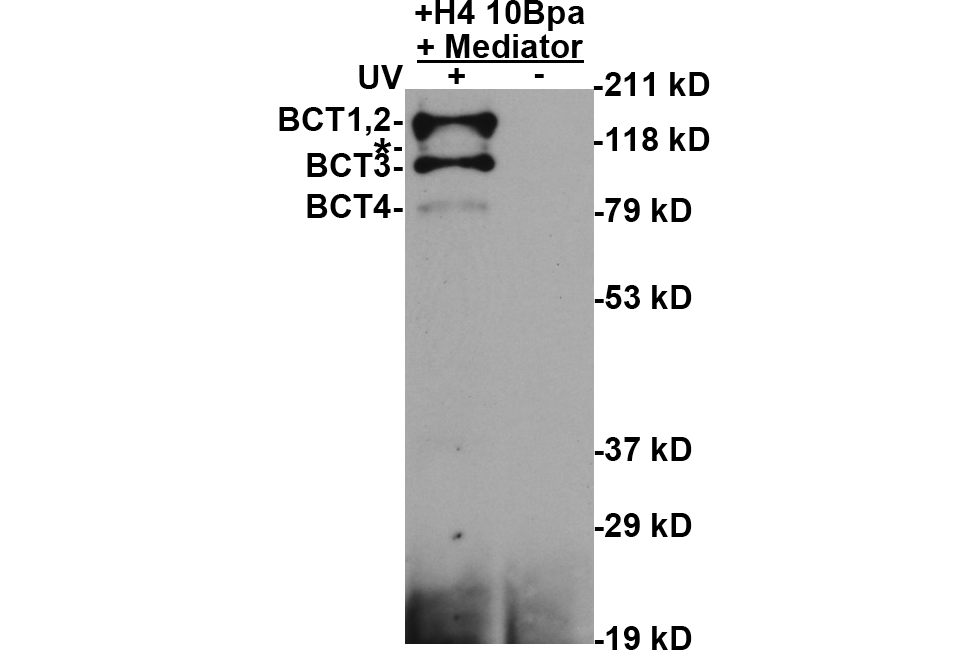

Supplement: Figure S2 — H4 10 Bpa cross-linking pattern resolved by 10% SDS-PAGE. SDS-PAGE blot probed with streptavidin poly-HRP to detect biotinylated peptide cross-linked to Mediator subunits resolved on 10% SDS-polyacrylamide. The high molecular weight signals are labelled as previously described (Fig. 2-A). (TIF) [file pone.0038416.s002.tif]

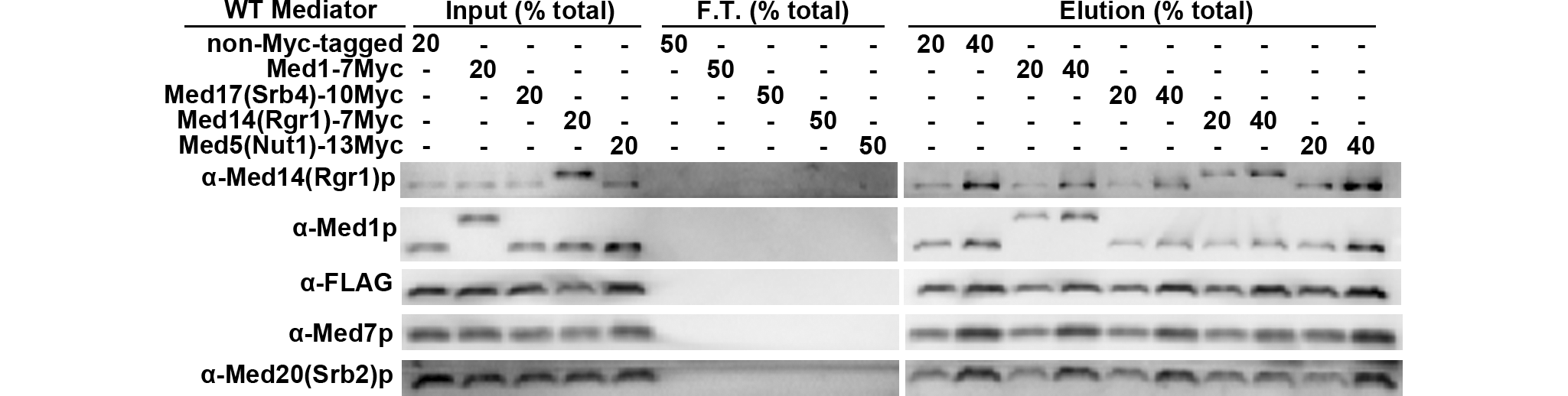

Supplement: Figure S3 — Myc -tagging does not interfere with the binding between Mediator complex and H4 10 Bpa peptide. Western blot analysis of a histone tail binding experiment in which each indicated Mediator species (∼3 nM) was mixed with H4 10 Bpa (4 µM) as the input. The basic steps and layout of the analysis were as described earlier (Fig. 1). (TIF) [file pone.0038416.s003.tif]
