# Supplementary material for: Med5(Nut1) and Med17(Srb4) Are Direct Targets of Mediator Histone H4 Tail Interactions
Source: PLoS One. 2012 Jun 5;7(6):e38416. doi: 10.1371/journal.pone.0038416 (PMC3367926; doi:10.1371/journal.pone.0038416)
Supplement: Table S1 — Yeast strains used in this study. (DOCX) [file pone.0038416.s004.docx]

| **Name** | **Genotype** | **Source** |
| --- | --- | --- |
| SHY349 | MATα *ade2Δ::hisG his3Δ300 leu2Δ0 lys2Δ0 met15Δ0 trp1Δ63 ura3Δ0* *MED18(SRB5)::MED18(SRB5)-3FLAG-KAN^R^* | [42] |
| yZL1 | yLM35; *MED1::MED1-7MYC-HIS3* | This study |
| yZL2 | yLM35; *MED17(SRB4)::MED17(SRB4)-10MYC-HIS3* | This study |
| yZL3 | yLM35; *MED14(RGR1)::MED14(RGR1)-7MYC-HIS3* | This study |
| yZL4 | yLM35; *MED5(NUT1)::MED5(NUT1)-13MYC-HIS3* | This study |
| #15489 | MATa *his3Δ1 leu2Δ0 lys2Δ0 ura3Δ0 med1::KAN^R^* | [45] |
| yLM79 | #15489; *MED18(SRB5)::MED18(SRB5)-3FLAG-NAT^R^* | This study |
| #15385 | MATa *his3Δ1 leu2Δ0 lys2Δ0 ura3Δ0 med9(cse2)::KAN^R^* | [45] |
| yZL15 | #15385; *MED18(SRB5)::MED18(SRB5)-3FLAG-NAT^R^* | This study |
| #14518 | MATa *his3Δ1 leu2Δ0 lys2Δ0 ura3Δ0 med5(nut1)::KAN^R^* | [45] |
| yLM74 | #14518; *MED18(SRB5)::MED18(SRB5)-3FLAG-NAT^R^* | This study |
| DY2694 | MATα *ade2 can1 his3 leu2 trp1 ura3 rgr1(med14)-Δ2::LEU2* | D.J. Stillman |
| yZL14 | DY2694; *MED18(SRB5)::MED18(SRB5)-3FLAG-NAT^R^* | This study |
| DY1876 | *MATa ade2 can1 leu2 trp1 ura3 med16(sin4)::TRP1* | D.J. Stillman |
| yLM61 | DY1876; *MED18(SRB5)::MED18(SRB5)-3FLAG-NAT^R^* | This study |
| yZL13 | yLM74*; med1::HIS3* | This study |

Table S1 Yeast strains used in this study
